# Effect and mechanism of human umbilical cord mesenchymal stem cells in treating allergic rhinitis in mice

**DOI:** 10.1038/s41598-020-76343-4

**Published:** 2020-11-09

**Authors:** Xiao-li Kan, Xing-hua Pan, Jing Zhao, Jie He, Xue-min Cai, Rong-qing Pang, Xiang-qing Zhu, Xian-bao Cao, Guang-ping Ruan

**Affiliations:** 1 Kunming Key Laboratory of Stem Cell and Regenerative Medicine, 920th Hospital of the PLA Joint Logistics Support Force, Kunming, 650032 Yunnan China; 2 Stem Cell and Immune Cell Biomedical Techniques and Integrated Engineering Laboratory of State and Regions, Kunming, Yunnan China; 3 Cell Therapy Technology Transfer Medical Key Laboratory of Yunnan Province, Kunming, Yunnan China; 4 Department of Otorhinolaryngology, Kunming First People’s Hospital, Kunming, Yunnan China

**Keywords:** Biological techniques, Molecular biology, Stem cells

## Abstract

A model of allergic rhinitis (AR) in BALB/c mice was established and evaluated to provide experimental subjects for further research. Preparation of human umbilical cord mesenchymal stem cells (hUCMSCs), including isolation, expansion culture, passaging, cryopreservation, and preparation of cell suspensions, provided materials for experimental research and clinical treatment. The mouse AR model was established by ovalbumin (OVA) intraperitoneal injection and the nasal stimulation induction method, and the model had a good effect and high repeatability. GFP-labeled hUCMSCs had good effects and were stable cells that could be used for tracking in animals. Transplantation of hUCMSCs by intraperitoneal and tail vein injections had a specific effect on the AR model of mice, and tail vein injection had a better effect. Tracking of hUCMSCs in vivo showed that the three groups of mice had the greatest number of hUCMSCs in the nose at week 2. The mouse AR model was used to evaluate the efficacy of hUCMSC transplantation via multiple methods for AR. The distribution of hUCMSCs in vivo was tracked by detecting green fluorescent protein (GFP), and the treatment mechanism of hUCMSCs was elucidated. This study provides technical methods and a theoretical basis for the clinical application of hUCMSCs.

## Introduction

Allergic rhinitis (AR) is a very common type of disease in the respiratory system that is caused mostly by type I allergic reactions in the nasal mucosa, which mainly manifest as paroxysmal repeated sneezing and a runny or stuffy nose; symptoms usually occur for 2 consecutive days or more and last more than 1 h per day. It often causes severely impaired olfactory function. The disease is also often called allergic rhinitis, nasal allergies and hay fever in publications. AR is involved in systemic inflammatory processes and is associated with inflammatory diseases such as asthma, sinusitis, and allergic conjunctivitis^1^. AR reduces quality of life by affecting sleep, learning, work efficiency and social life, resulting in higher social and healthcare costs. Due to its high prevalence and impact on quality of life, it has been listed as a major chronic respiratory disease. At the same time, the disease is also one of the main causes of other related diseases, such as anxiety and depression.

In recent years, the prevalence of AR has been increasing each year^2^, affecting a large number of people, and it has become one of the most common global chronic health problems. The initial allergen exposure and sensitization of AR involves antigen-presenting cells, and the production of T and B lymphocytes was associated with allergen-specific T cells and allergen-specific T cell-related IgE antibodies^3^. After re-exposure to related allergens, mast cell IgE forms cross-links and releases allergic molecules such as histamine, which immediately causes nasal symptoms. Within a few hours, there is infiltration of inflammatory cells, especially Th2 lymphocytes, eosinophils, and basophils, into the nasal mucosa, leading to an enhanced allergic reaction^4^. Therefore, the preventive treatment of AR mainly includes avoiding allergens, drug treatment and immunotherapy. Current pharmacological options include oral and intranasal antihistamines, intranasal corticosteroids, oral and intranasal decongestants, oral and intranasal anticholinergics, and leukotriene receptor antagonists^5^. Second-generation anti-rhinitis drug amines and intranasal corticosteroids are still the main drugs, and there are practical guidelines recommending intranasal corticosteroids as first-line drugs for the treatment of moderate to severe AR. The goal of treatment is to reduce or eliminate current symptoms while preventing future damage and long-term complications. Appropriate treatment options should take into account the minimization of side effects and enable patients to maintain a normal lifestyle^6^. However, currently, there are many problems in treatment, such as poor drug compliance, adverse reactions, and persistent clinical symptoms, and no drugs or technologies have been found that can completely treat this type of disease. Therefore, it is important to find a more effective treatment plan.

Mesenchymal stem cells (MSCs) are a general term for adult mesenchymal stem cells capable of differentiating into various mesenchymal lines in vitro. They can be routinely isolated from tissues and easily stored in vitro. MSCs have the following main characteristics: (1) they exists in almost any tissue and have diverse tissue sources, including bone marrow^7^, peripheral blood, umbilical cord blood, adipose tissue, and dental pulp; (2) they have strong self-renewal and differentiation potential^8^; they can differentiate into a variety of lineages, such as adipocytes, chondrocytes, bone cells^9^, ectodermal cells (such as neuronal cells), and endoderm cells (such as liver cells and pancreatic cells^10^); (3) their expression of cell surface antigens such as CD11b, CD14, CD34, CD45 and human leukocyte antigen-DR (HLA-DR) are negative, while that of CD73, CD90, CD105, and other antigens are positive; (4) they have immunomodulatory characteristics, such as low MHC I and MHC II expression and reduced allograft and self-protection effects caused by costimulatory molecules such as CD80, CD40, and CD86; (5) they have the ability to change the host immune environment and affect the secretion of anti-inflammatory molecules (such as cytokines and antibodies). The regulation of the levels of IL-4 and IFN-γ in T1 immune response effector T cells reduces the response of the disease in terms of the conversion of the Th2 response (T helper cells). Among MSCs from many sources, human umbilical cord mesenchymal stem cells (hUCMSCs) have greater advantages than those of other stem cells^11^. In comparison, tissue separation is relatively easy when using raw umbilical cord, and there is no obvious risk or moral constraints for donors; furthermore, hUCMSCs have an enhanced ability to self-renew and differentiate and have lower immunogenicity^12^.

The development of effective therapies for AR depends on the implementation of effective methods to prevent further inflammatory lesions in the body and promote the recovery of tissue functions. Stem cell transplantation provides a novel and exciting treatment method^13^. In fact, the clinical application of hUCMSCs has great prospects for medicine (such as cell transplantation), pharmaceutical science (such as controlled drug delivery) and biological science (such as tissue engineering). In addition to promoting tissue repair and other characteristics, hUCMSCs have shown a great capacity for effective immune regulation in vivo and in vitro. Therefore, hUCMSCs are used as an immunomodulatory means for the treatment of autoimmune diseases, graft-versus-host disease (GvHD) and allogeneic rejection. Numerous studies have reported promising results in the treatment of GvHD, multiple sclerosis, and Crohn's disease. Similarly, there have been reports of the use of MSCs in the treatment of AR^14^. A large number of preclinical experiments and clinical trials are currently underway to study the role and mechanism of hUCMSCs as a form of cellular immunotherapy^15^.

The research purpose of this project is to isolate and culture hUCMSCs by the repeated adherence method and to determine their quality to obtain hUCMSCs that meet the application standards and can be used in clinical practice. Additional aims are to establish an efficient and stable mouse AR model for subsequent research on diseases, to explore the effects and mechanisms of hUCMSC transplantation on the AR model in mice and to provide technical methods and a theoretical basis for hUCMSC application in clinical treatment.

## Materials and methods

### Experimental animals and cells

The BALB/c mice used in this experiment were provided by Hunan Slake Jingda Experimental Animal Co., Ltd. The laboratory animal certificate number was 43004700044790, and the mice were maintained in the Experimental Animal Center of the 920th Hospital of the People's Liberation Army Joint Service and Support Force. All animals were clean. All animals were raised in accordance with the conventional methods in the appropriate environment, and all animal experiments were performed in accordance with the relevant guidelines for animal experiments. HUCMSCs were obtained from the obstetrics department of the 920th Hospital of the People’s Liberation Army’s Joint Security Forces. Umbilical cords of healthy term fetuses were collected under sterile conditions. The mothers had no history of infectious diseases or genetic diseases, and the fetuses had no congenital diseases. The study was approved by the Hospital Ethics Committee under ethics number 2018017. A sufficient amount of hUCMSCs was obtained and cultured from the collected human umbilical cord and stored in a liquid nitrogen tank for future use. We confirmed that informed consent was obtained from all participants and/or their legal guardians.

### Establishment of the mouse AR model

Fifteen healthy 4- to 6-week-old BALB/c female mice were selected, all of which were normally kept in a clean, specific pathogen-free (SPF) environment. All mice were acclimatized for one week before the experiment began, and thereafter, they were randomly divided into model groups A and B and control group N, with 5 mice in each group. Follow-up experiments were performed according to the group. Animal discomfort, pain and suffering were avoided or reduced during the experiment. For group A, at the same time each day on days 0, 7, and 14 after grouping, basic sensitization was performed using a 1 ml sterile syringe, and each mouse was injected intraperitoneally with 200 µl adjuvant suspension containing 25 µg ovalbumin (OVA)/2 mg Al(OH)_3_; for group B, at the same time each day on days 0, 7, and 14 after grouping, basic sensitization was performed using a 1 ml sterile syringe, and 200 μl of 50 µg OVA in 2 mg Al(OH)_3_ adjuvant suspension was injected into the abdominal cavity of each mouse; for group N, at the same time each day on days 0, 7, and 14 after grouping, each mouse was injected intraperitoneally with 200 µl of saline using a 1 ml sterile syringe. The other treatment measures were the same for all groups, and attention was paid to the state of each mouse. For groups A and B, 20 μl (10 μl in each nostril) of the current OVA challenge solution was given to each mouse at the same time every day on days 15–21 for the nasal drip challenge. Immediately after the nasal drip challenge was performed, the nose and the occurrence of a runny nose and sneezing in the mice were observed and recorded (continuous scratching of the nose was regarded as a single reaction). For group N, on days 15–21, 20 μl of physiological saline (10 μl in each nostril) was administered to each mouse at the same time every day for nasal stimulation. Immediately after the nasal drip challenge was performed, the occurrence of a runny nose, scratching of the nose, and sneezing was observed and recorded. Continuous scratching of the nose was regarded as a single reaction. All mice were sacrificed within 24 h of the last nasal stimulation procedure, and materials were collected for subsequent experiments.

### Behavioral observation

The general symptoms of the mice were observed. From the first day of modeling, the behavior, morphology, and feeding of the mice were observed, and the occurrence of scratching, sneezing and runny nose in the mice were observed within 15 min after each nasal challenge. The total score was calculated. If the total score exceeded 5 points, the model was successfully created (see Table 1). The groups were compared.

**Table 1:** General symptom scores of the mice.

Item	1 point	2 points	3 points
Scratching of the nose	Flicking of the nose several times with one claw	Repeated scratching of the nose with both claws	Friction around the nose
Sneezing	1–3	4–10	> 11
Runny nose	Snot flowing into the front nostril	Snot flowing over the front nostril	Runny nose

### Collection of laboratory animal tissues and blood samples

Within 24 h of the last treatment, peripheral blood was collected from the mice in each group by removing the eyeballs, and the blood was left at room temperature for approximately 2 h, centrifuged at 860×*g* for 10 min in a 4 °C thermostatic centrifuge and then pipetted. The upper serum was carefully removed and stored in a refrigerator at − 80 °C for later use. The spleens of each group of mice were placed in EPPCs treated with DEPC water, which were autoclaved, quickly frozen in liquid nitrogen, and stored in a − 80 °C freezer. The nasal breathing zone mucosa was preserved, fixed in 4% paraformaldehyde solution, stored at room temperature, and used for HE staining of tissue sections.

### HE staining and observation of nasal mucosa tissue sections

The mucous membrane of the nasal breathing zone was fixed with 4% paraformaldehyde solution, paraffin embedded and dewaxed. The sections were soaked twice in xylene for 20 min and then soaked in absolute ethanol for 5 min. Then, the samples were soaked in 75% alcohol for 5 min and rinsed with tap water. After that, hematoxylin eosin staining was routinely performed: the sections were soaked in hematoxylin staining solution for 5 min, rinsed with tap water once, placed into differentiation solution to induce differentiation, and then rinsed with tap water. The sections were then rinsed with tap water, soaked and dehydrated in 85% and 95% alcohol for 5 min each and then soaked in eosin for 5 min. The dehydration and sealing procedures were then performed. The slices were soaked in anhydrous ethanol for 5 min three times each for dehydration and then soaked twice in xylene for 5 min. The sections were observed carefully, and image acquisition and analysis were performed under a light microscope. The main concern was the observation of the infiltration of inflammatory cells and histomorphological changes.

### Detection of IL-4 and IFN-γ in mouse serum by ELISA

The serum samples of each group of mice that were previously stored were diluted as needed, and the concentrations of IL-4 and INF-γ in the serum of the mice were measured using an ELISA kit. The instructions provided with each ELISA kit were strictly followed. The OD value was detected at 450 nm using a microplate reader within 5 min after the reaction. The standard concentration represented the abscissa, and the OD value represented the ordinate. Regression fitting was performed by computer software to generate a standard curve. Regression analysis was used to obtain the best standard curve. The OD value of each sample was compared to the standard curve to obtain the corresponding IL-4 and IFN-γ concentrations in mouse serum.

### Detection of the total protein content in serum by using the BCA method

A small number of mouse serum samples from each group were diluted at the required ratio, and a BCA protein quantification kit was used to perform the quantitative determination of total serum protein according to the instructions.

### Determination of the transcription levels of IL-4, IL-6, IL-10 and IFN-γ mRNA in mouse spleen tissue by PCR

The spleen samples of each group of mice were refrigerated at − 80 °C, and then they were ground into small tissue pieces using a mortar and liquid nitrogen. The ground tissue was placed in a pretreated EP tube, to which 500 μl of TRIZOL reagent was added, and the tube was shaken well and incubated at room temperature for 10 min for pyrolysis; then, 100 μl of chloroform was added, and the tube was shaken well for 30 s until red and white layers formed. The tube was centrifuged at 13,600×*g* for 10 min at 4 °C. The upper aqueous phase was pipetted into a new EP tube, to which 250 μl of prerefrigerated isopropanol was added, and the tube was mixed and placed on ice for 10 min. The tube was then centrifuged at 13,600×*g* for 10 min at 4 °C. The supernatant was discarded, 500 μl of prechilled 75% ethanol was added, and the EP tube was gently shaken to resuspend the pellet. The tube was centrifuged at 13,600×*g* at 4 °C for 5 min, and the supernatant was discarded; the lid was left open to ventilate the excess ethanol (without overdrying; otherwise, the solubility of RNA will be greatly reduced). Approximately 30–50 μl DEPC water was added to dissolve the total RNA sample. The A260/A280 ratio of the sample was measured by a DNA/RNA concentration detector, and the ratio was between 1.8–2.0, which met the requirements for the RNA samples; the RNA sample concentration was recorded. The extracted RNA samples were reverse-synthesized into cDNA using a reverse transcription kit (PrimeScript™ RT reagent Kit with gDNA Eraser, purchased from TAKARA), and real-time quantitative PCR was performed using SYBR Premix Ex Taq II (purchased from TAKARA). A Life ECO DNA amplification instrument and PCR analyzer were used for the experimental procedure and data analysis. All primer sequences are shown in Table 2.

**Table 2:** Primer sequences.

Gene sequence number	Gene base sequence	Sequence length (bp)
IL-4 (NR_027491.1)	Forward 5′-GGTCTCAACCCCCAGCTAGT-3′	20
	Reverse 5′-GCCGATGATCTCTCTCAAGTGAT-3′	23
IL-6 (M24221.1)	Forward 5′-CCAAGAGGTGAGTGCTTCCC-3′	20
	Reverse 5′-CTGTTGTTCAGACTCTCTCCCT-3′	22
IL-10 (NM_010548.2)	Forward 5′-GCTCTTACTGACTGGCATGAG-3′	21
	Reverse 5′-CGCAGCTCTAGGAGCATGTG-3′	20
IFN-γ (S69336.1)	Forward 5′-ATGAACGCTACACACTGCATC-3′	21
	Reverse 5′-CCATCCTTTTGCCAGTTCCTC-3′	20
β-Actin (AY355144.1)	Forward 5′-GGCTGTATTCCCCTCCATCG-3′	19
	Reverse 5′-CCAGTTGGTAACAATGCCATGT-3′	22

### Culture, identification and GFP labeling of human umbilical cord mesenchymal stem cells

The umbilical cord of a healthy full-term fetus was collected under sterile conditions in the delivery room of the hospital's maternity department. To remove the residual blood, the collected umbilical cord was repeatedly washed with normal saline. Then, the tissue was cut to a size of approximately 1 mm^3^ and inoculated into a 175 cm^2^ cell culture flask, to which 20 ml of complete medium containing 10% fetal bovine serum was added; the flask was cultured in a high humidity incubator at 37 °C in 5% CO_2_. The fluid exchange operation was performed for the first time after 5 days, and the cell growth was observed to reach 80% after 7 days. After confluence was reached, passaging was performed at a ratio of 1:2. After the primary cells were cultured for approximately 1 week and passaged to the 3rd–5th generation, the morphology and growth of the cells were observed and photographed under an inverted phase contrast microscope at each stage.

HUCMSCs in the P4 generation at a confluence of more than 80% were selected. Next, 5 μl FITC-labeled CD45 and CD90 and PE-labeled CD34, CD105, and CD73 antibodies were added to each tube. The negative controls and isotype controls were set up and incubated for 30 min in the dark. Flow cytometry was used to analyze the positive expression ratios of the surface antigens.

Analysis of the ability of hUCMSCs to induce adipogenic, osteogenic and chondrogenic differentiation in vitro:

Transfection of a GFP-encoding lentivirus was performed when the cells were growing well and the degree of confluence reached at least 60%. Some of the cells were selected and trypsinized, and the GFP-positive rate of the hUCMSCs was analyzed by flow cytometry.

### Evaluation of the efficacy and mechanism of hUCMSCs

#### Treatment of hUCMSCs

Forty healthy female BALB/c mice aged 4–6 weeks were randomly divided into the healthy control group N (n = 10), the AR model control group M (n = 10), the AR model treatment group A (n = 10) and the AR model treated group B (n = 10). The four groups of mice were normally kept in an SPF environment, in which the N group was used as a healthy control group without any special treatment; the M group was used as a model control group. Twenty-five micrograms OVA/2 mg Al(OH)_3_ adjuvant was administered in a volume of 200 μl via intraperitoneal injection for basic sensitization once a week for three weeks, and then OVA stimulation solution was continuously administered via a nasal drip until the 37th day without special treatment. Group A served as the AR model and was subjected to intraperitoneal injection. The model operation was the same as that used for the M group, but starting on the 22nd day of the experiment, each mouse was injected intraperitoneally with 2.0 × 10^5^ cells/ml in 200 µl hUCMSC suspension for treatment once every 3 days until the 37th day of the experiment; a total of 6 treatments were performed. Group B was used as the AR model tail vein injection treatment group, and the modeling operation was also the same as that used for the M group; starting on the 22nd day of the experiment, each mouse was injected with an hUCMSC suspension with a concentration of 2.0 × 10^5^ cells/ml. Treatment was administered at a volume of 200 µl with the same frequency and duration as that used for the treatment of group A.

Preparation of the hUCMSC suspension for the experiment: The cells were trypsinized and collected into a 50 ml centrifuge tube. The cells were washed once with normal saline and then centrifuged at 400×*g* for 4 min; the supernatant was removed, and the cells were resuspended in normal saline and filtered with a cell filter to prevent cell clumping. The cells were observed under a microscope and counted, and the cell concentration was adjusted to 2.0 × 10^5^ cells/ml. The cells were kept on ice and used as soon as possible. The injection operation used a disposable 1 ml sterile syringe. The injection site was disinfected with an alcohol-soaked cotton ball before the injection, and the eye was compressed at the position of the needle to prevent extravasation of the injected liquid and promote liquid absorption.

#### Evaluation of the efficacy of hUCMSCs

From the beginning to the end of treatment, the behavior, morphology, and feeding of AR model mice were observed in each group every day, and the occurrence of nose scratching, sneezing, and runny nose in each group of mice was recorded within 15 min after each treatment, after which the total score was calculated. The groups were compared; on the 38th day, the nasal mucosa of the mice were stored in 4% paraformaldehyde, and the conventional tissue sections were stained with HE in the later stage to observe the changes in the tissue structure of the mice in each group; ELISA was also used. The concentrations of IL-4 and IFN-γ in the serum of the mice in each group were measured by ELISA; the total protein content in the serum of the mice in each group was measured by the BCA method. The IL-4, 6, and 10 and IFN-γ mRNA transcription levels were also measured to evaluate the effect of hUCMSC transplantation on the mouse AR model.

#### Exploration of the therapeutic mechanism of hUCMSCs

Thirty-six healthy female BALB/c mice aged 4–6 weeks were randomly housed in an SPF environment without specific pathogens and randomly divided into three groups. Group A was the nasal drip group (n = 12); group B was the intraperitoneal injection group (n = 12); and group C was the tail vein injection group (n = 12). On the same day, hUCMSCs labeled with GFP with good growth status were selected from the P5 generation, and the cells were collected to generate the hUCMSC suspensions. Mice in group A received 200 µl/head of the hUCMSC suspension via nasal drip; 100 µl/head of the hUCMSC suspension was injected intraperitoneally into mice in group B. The mice in group C were injected with 200 µl of the hUCMSC suspension. Seven days after the operation, the materials were collected once, and the nasal mucosa, lungs, ovaries, and hearts of the mice were collected. On the 14th and 21st days, samples were taken once, and the nasal mucosa of the mice were collected. Samples were obtained from 4 mice in each group. The organs and part of the nasal mucosa were fixed in 4% paraformaldehyde for the single labelling of tissue sections with green fluorescence. GFP-labeled hUCMSCs were observed in the mice. The other part of the nasal mucosa was placed in an EP tube. The tissue was cut into pieces of approximately 1 mm^3^ with ophthalmic scissors and then transferred into a 50 ml centrifuge tube. An approximately fivefold volume of 0.1% type II collagenase was added, and the tube was placed at 37 °C in a constant temperature shaker for 60 min. The tissue was filtered with a cell filter and transferred to a new centrifuge tube. A volume of 45 ml of normal saline was added, and the tube was centrifuged at 400×*g* for 10 min. The supernatant was discarded, and the cells were resuspended by adding the required amount of PBS. Then, we observed whether the tissue was digested into single cells under the microscope, and we counted the cells and analyzed the proportion of green fluorescent cells by flow cytometry.

### Statistical analysis

The data obtained in this experiment are expressed as the mean ± standard deviation of the significance of the differences between the experimental groups. Using IBM SPSS Statistics v24 statistical software, the tests were performed using repeated measures and analysis of variance. The LSD-t test was used to compare the differences when the variance was equal. When the variances were unequal, the Tamhane T2 method was used to make a pairwise comparison, and p < 0.05 was used as the index of a significant difference.

### Ethics approval and consent to participate

The experimental protocols were approved by the Experimental Animal Ethics Committee of the 920th Hospital of the PLA Joint Logistics Support Force.

## Results

### AR model establishment and evaluation results

#### General status observation

**Figure 1:**
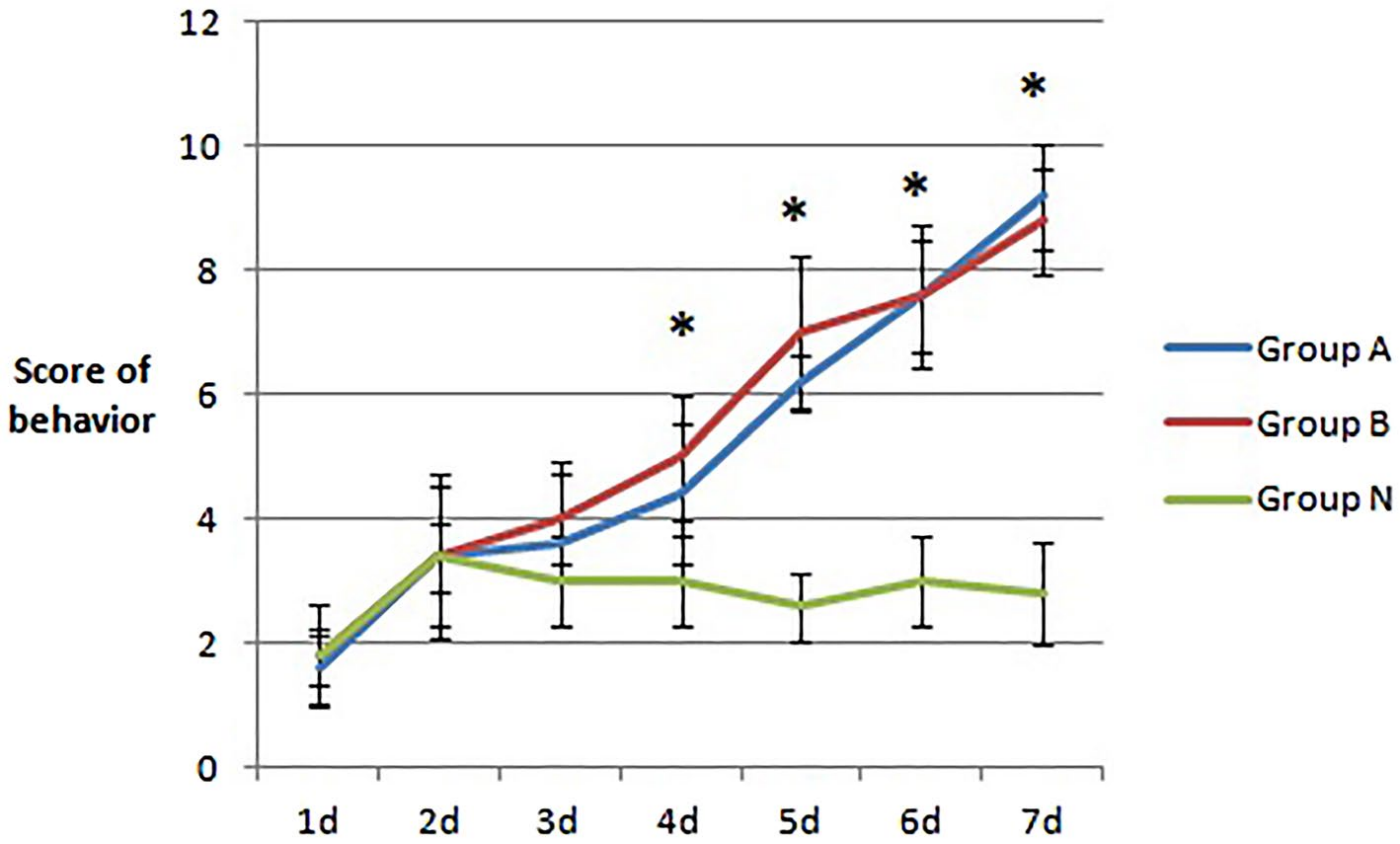
Changes in the total behavioral scores of three groups of mice after several days of stimulation. Compared with group N at 4, 5, 6, and 7 days, there were significant differences between group A, B and group N (*P < 0.05), but there was no significant difference between groups A and B (P > 0.05).

During the experimental observation, no death occurred in the three groups of mice. Compared with those in the N group, the total behavioral scores of the mice in the A and B groups were significantly increased after the nasal drip, sneezing, and runny nose (see Fig. 1); starting on the 4th day after the nasal drip, the total behavioral scores of the A and B groups were significantly higher than those of the N group (p < 0.05). Starting on the 5th day, the total behavioral scores in the A and B groups were all greater than 5 points, indicating that the mouse models in the A and B groups were successfully modeled.

#### Structural changes of the nasal mucosa

**Figure 2:**
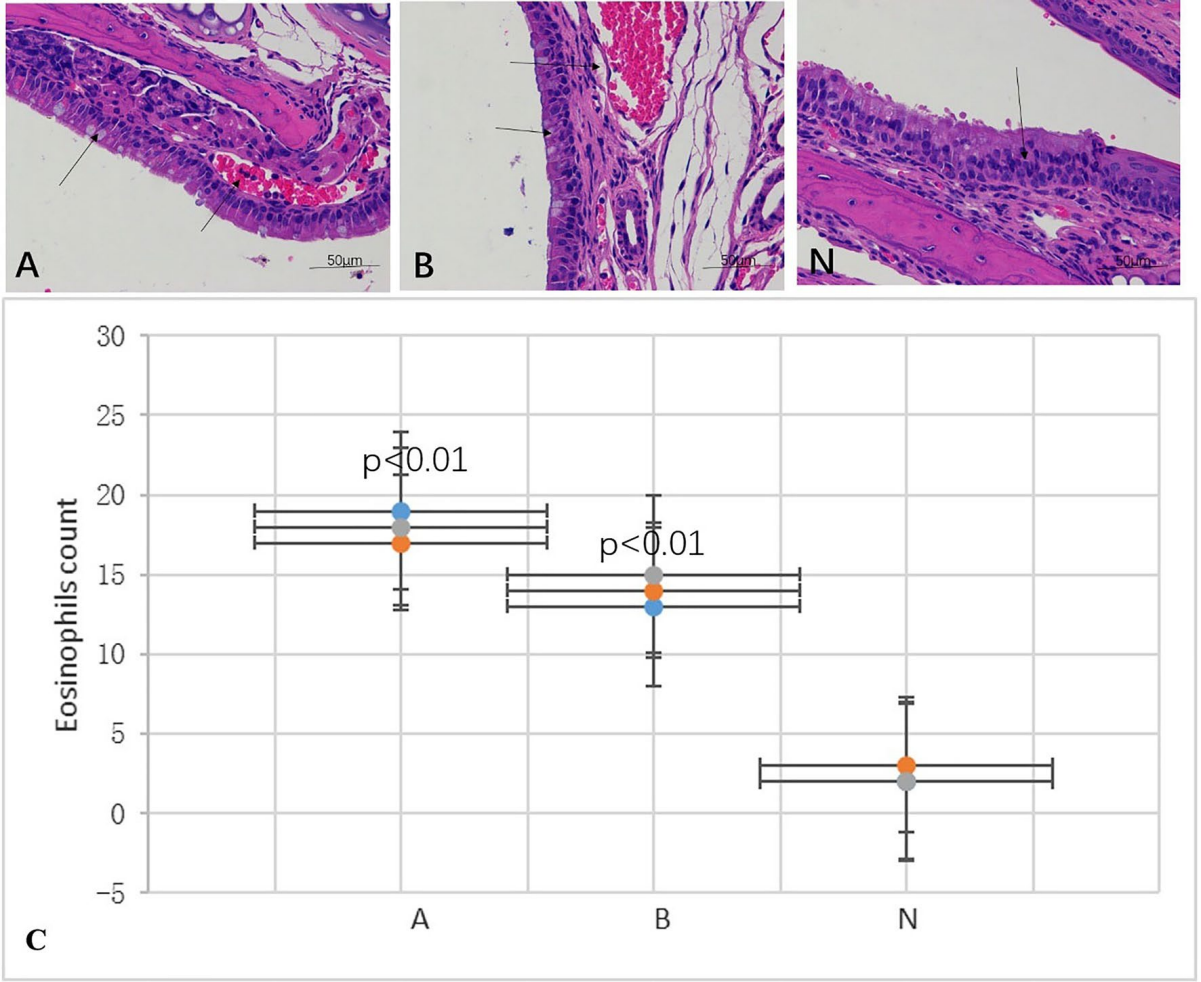
HE staining of mouse nasal mucosa tissue sections. N represents the normal nasal mucosa. (**A**) and (**B**) are the model groups, and a large number of eosinophils had infiltrated, as shown by the black arrow. Eosinophils counts were showed in (**C**).

The results of HE staining showed that the normal nasal mucosa should be pseudostratified ciliated columnar epithelium, which is mainly composed of ciliated columnar epithelial cells and goblet cells with a small number of blood vessels and no eosinophils infiltration and changes in blood vessels (see Fig. 2N). In the model group, the cilia detached, the small blood vessels proliferated and expanded, and a large number of eosinophils infiltrated, as shown by the black arrow. The number of submucosal glands increased (see Fig. 2A,B). Eosinophils counts were showed in Fig. 2C.

#### Serum IL-4 and INF-γ concentrations

**Figure 3:**
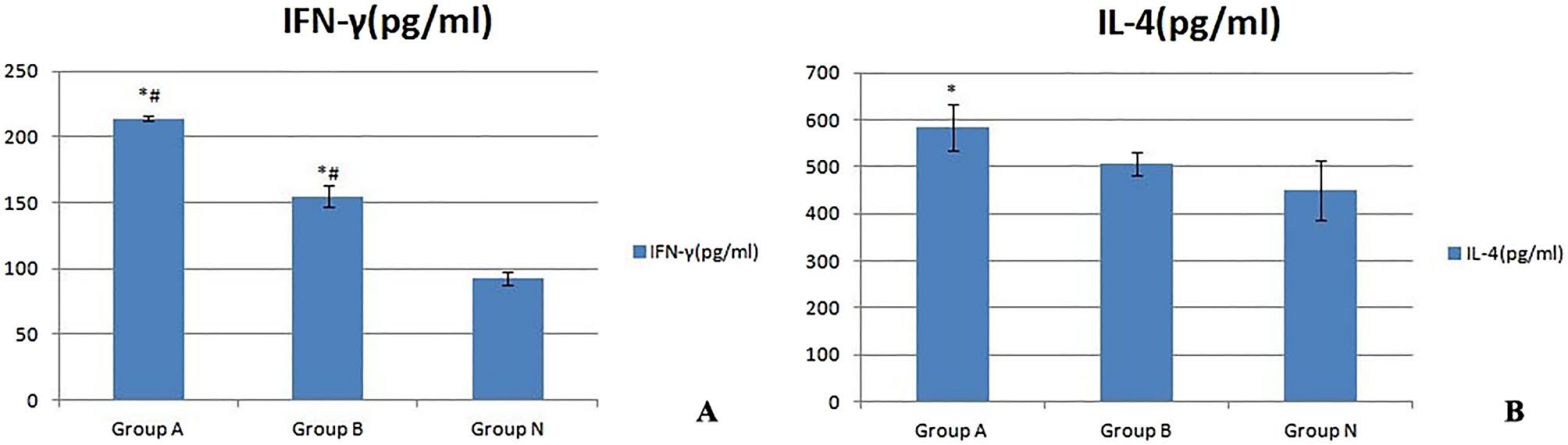
Serum IL-4 and INF-γ concentrations of the three groups of mice. Compared with those in the N group, the serum IFN-γ concentrations in the A and B groups were significantly increased (*p < 0.05), and the increase in the A group was more significant (^#^p < 0.05).

The results of the ELISA used to detect the peripheral serum IFN-γ and IL-4 concentrations are shown below (see Fig. 3). Compared with those in the N group, the serum IFN-γ concentrations in the A and B groups were significantly increased (*p < 0.05), and the increase in the A group was more significant (^#^p < 0.05). Compared with that in the N group, the serum IL-4 concentration in the A group increased significantly (*p < 0.05), but that in group B increased only slightly (p > 0.05); the difference between groups A and B was not significant (p > 0.05).

#### Total serum protein content of mice

**Figure 4:**
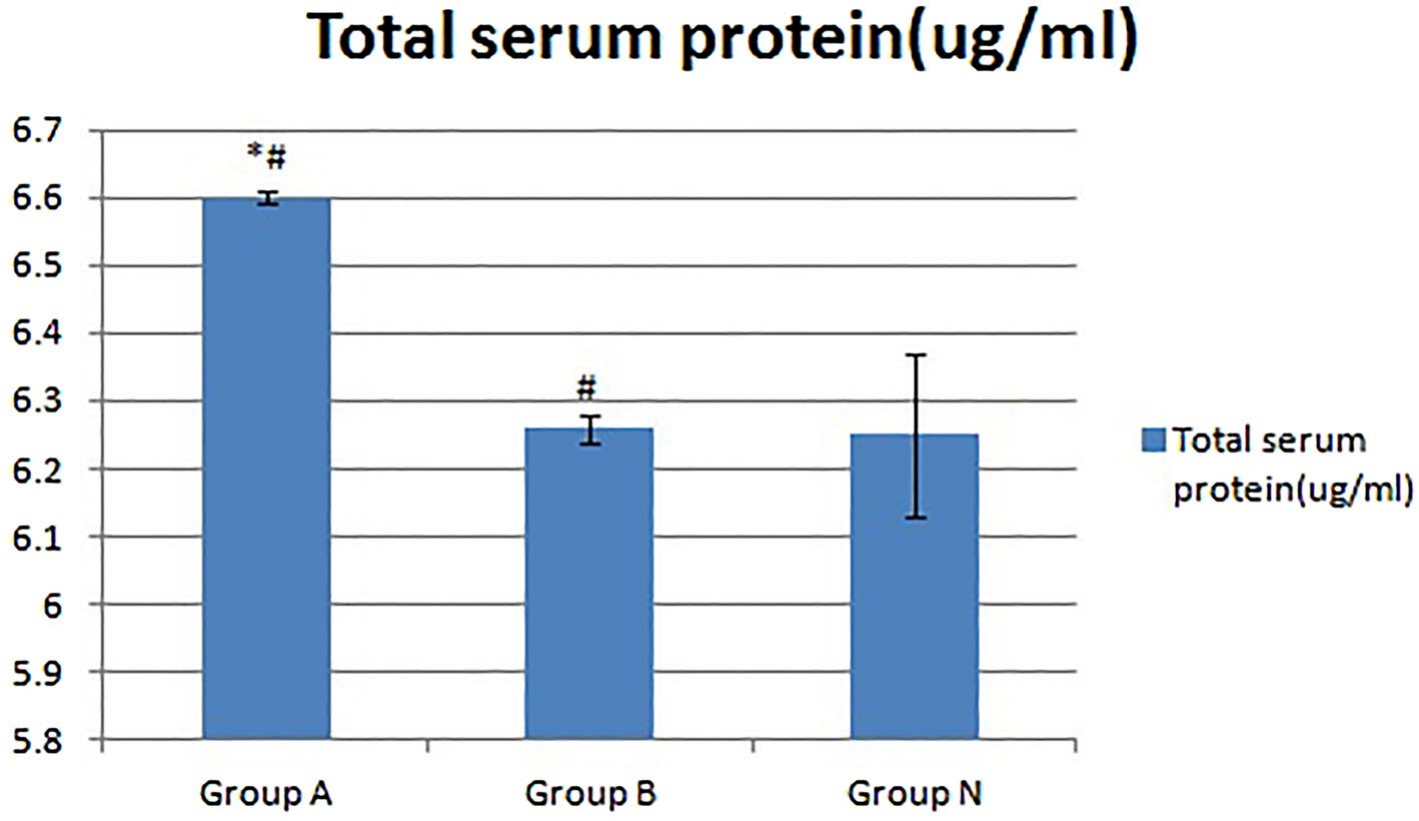
Changes in the serum total protein content of the three groups of mice. Compared with that in group N, the total serum protein content in group A increased significantly (*p < 0.05), but the total blood protein content in group B increased nonsignificantly (p > 0.05). Compared with that in group B, the total protein content in peripheral serum in group A also increased significantly (^#^p < 0.05).

The results of measuring the total serum protein content in the peripheral blood of mice by the BCA protein quantitative method are shown in Fig. 4. Compared with that of group N, the total serum protein content of group A increased significantly (*p < 0.05), but the total blood protein content of group B showed a smaller increase (p > 0.05). Compared with that of group B, the total protein content in peripheral serum of group A also increased significantly (^#^p < 0.05).

#### IL-4, IL-6, IL-10 and INF-γ mRNA transcription levels in mouse spleen tissue

**Figure 5:**
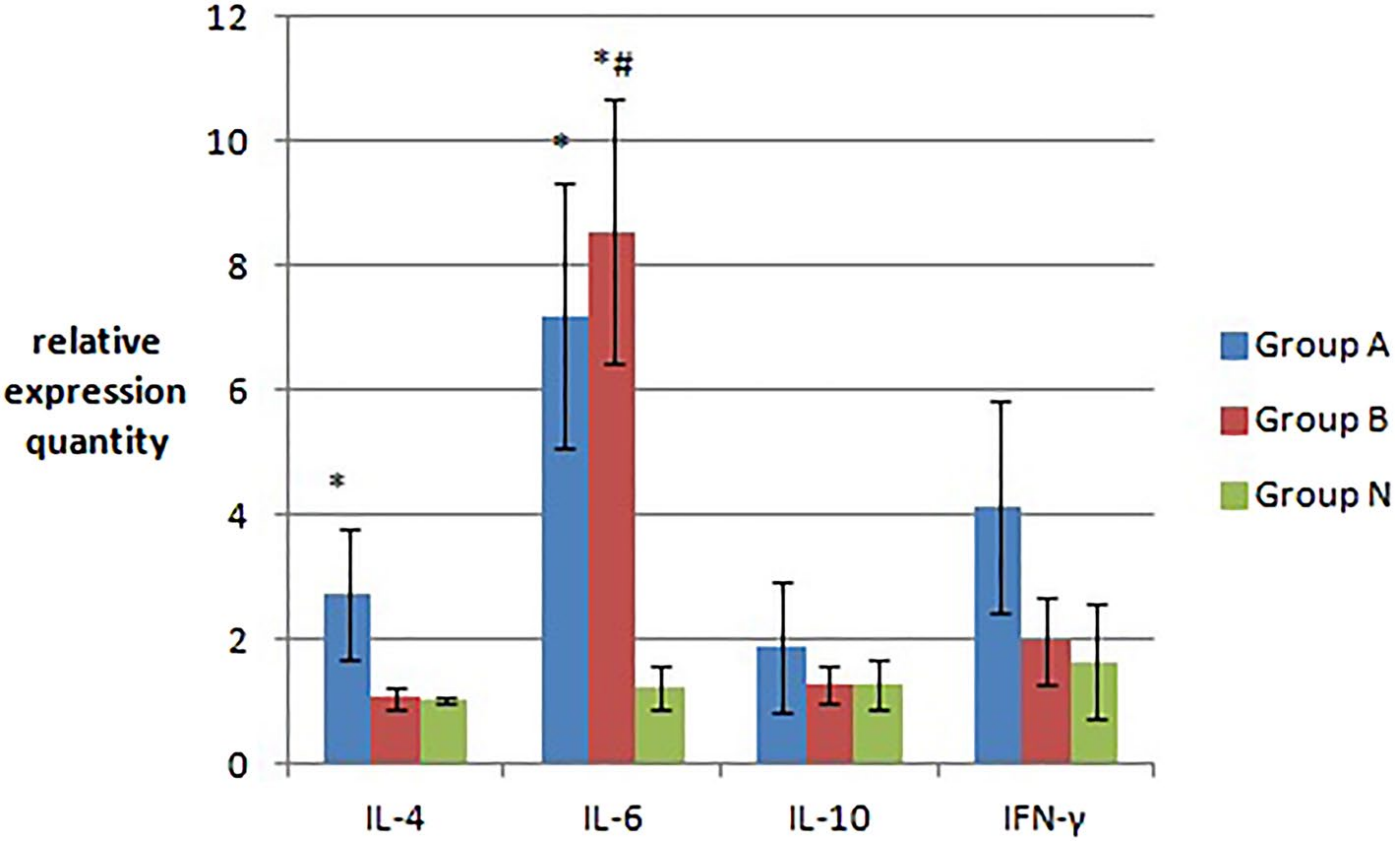
The transcript levels (relative expression quantity) of IL-4, IL-6, IL-10 and INF-γ mRNA in the spleen tissue of the three groups of mice. The IL-4 and IL-6 mRNA transcript levels in group A and group B increased significantly (*p < 0.05). The transcript levels of IL-6 mRNA were significantly increased in group B (^#^p < 0.05) compared with those in group A.

Real-time quantitative PCR was used to measure the transcription levels of IL-4, IL-6, IL-10 and IFN-γ mRNA in the spleen of mice. The results are shown in Fig. 5. Compared that of with group N, the mouse spleen tissue of groups A and B showed increased transcript levels of IL-4, IL-6, IL-10 and IFN-γ mRNA. However, the levels of IL-4 and IL-6 in group A and the IL-6 mRNA transcript levels of group B increased significantly (*p < 0.05). The mRNA transcript levels of the other factors increased only slightly (p > 0.05). The transcript levels of IL-6 mRNA were significantly increased in group B (^#^p < 0.05) compared with those in group A. The IL-4, IL-10 and IFN-γ mRNA transcript levels of group A were higher than those of group B, but the difference between groups A and B was not obvious (p > 0.05).

### Culture and identification of hUCMSCs

#### hUCMSC growth morphological observation

**Figure 6:**
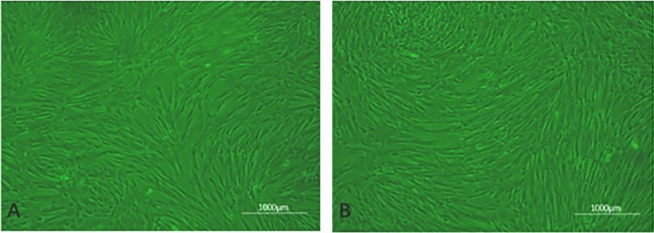
Morphological observation of hUCMSCs. (**A**) Shows the cell morphology of the P3 generation; (**B**) shows the cell morphology of P5 generation.

The primary cells were routinely cultured for approximately 1 week, and then three to four subcultures were generated. Observation under an inverted microscope showed that the hUCMSCs had a morphology typical of stem cells. After the third generation of hUCMSCs was cultured for 3 days, the cells were evenly scattered and had grown on the bottom wall of the culture flask, and the cells were uniform in shape with a long spindle shape and were fibroblast-like (Fig. 6A). When the cells were cultured until the fifth generation, the cells were still in a typical state, and when the cell density was high, they showed vortex-like growth characteristics (Fig. 6B).

#### hUCMSC surface antigen expression

**Figure 7:**
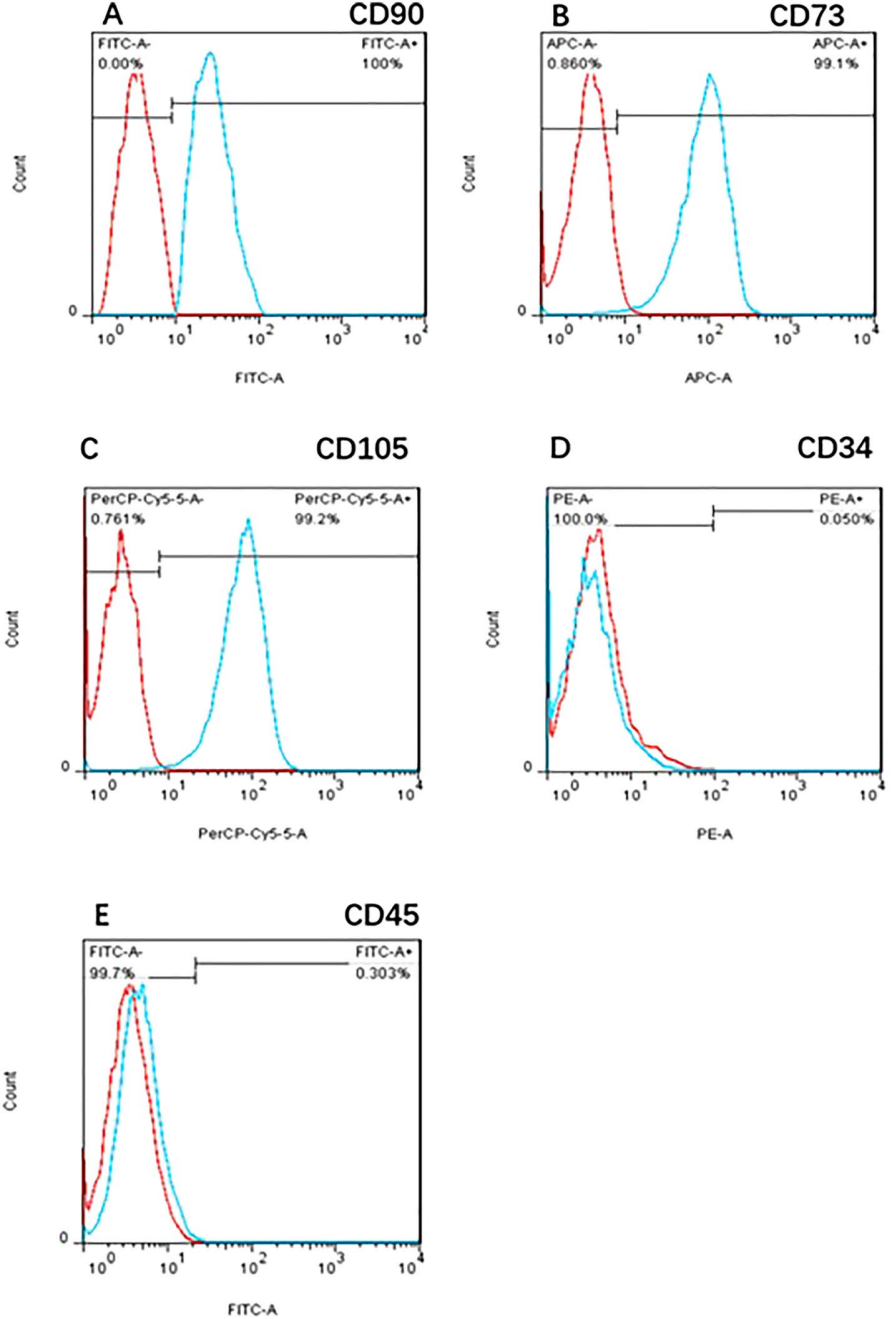
P4 hUCMSC surface antigen expression. Flow cytometry analysis of the surface antigens in 4th generation hUCMSCs showed that the positive expression ratios of CD90, CD73, and CD105 were 100.0%, 99.1%, and 99.2%, respectively, and the positive expression ratios of CD34 and CD45 were 0.05% and 0.30%, respectively.

Flow cytometry analysis of the 4th generation hUCMSC surface antigens showed that the positive expression ratios of CD90, CD73, and CD105 were 100.0%, 99.1%, and 99.2%, respectively, and the positive expression ratios of CD34 and CD45 were 0.05% and 0.30%, respectively (Fig. 7).

#### hUCMSC differentiation results

**Figure 8:**
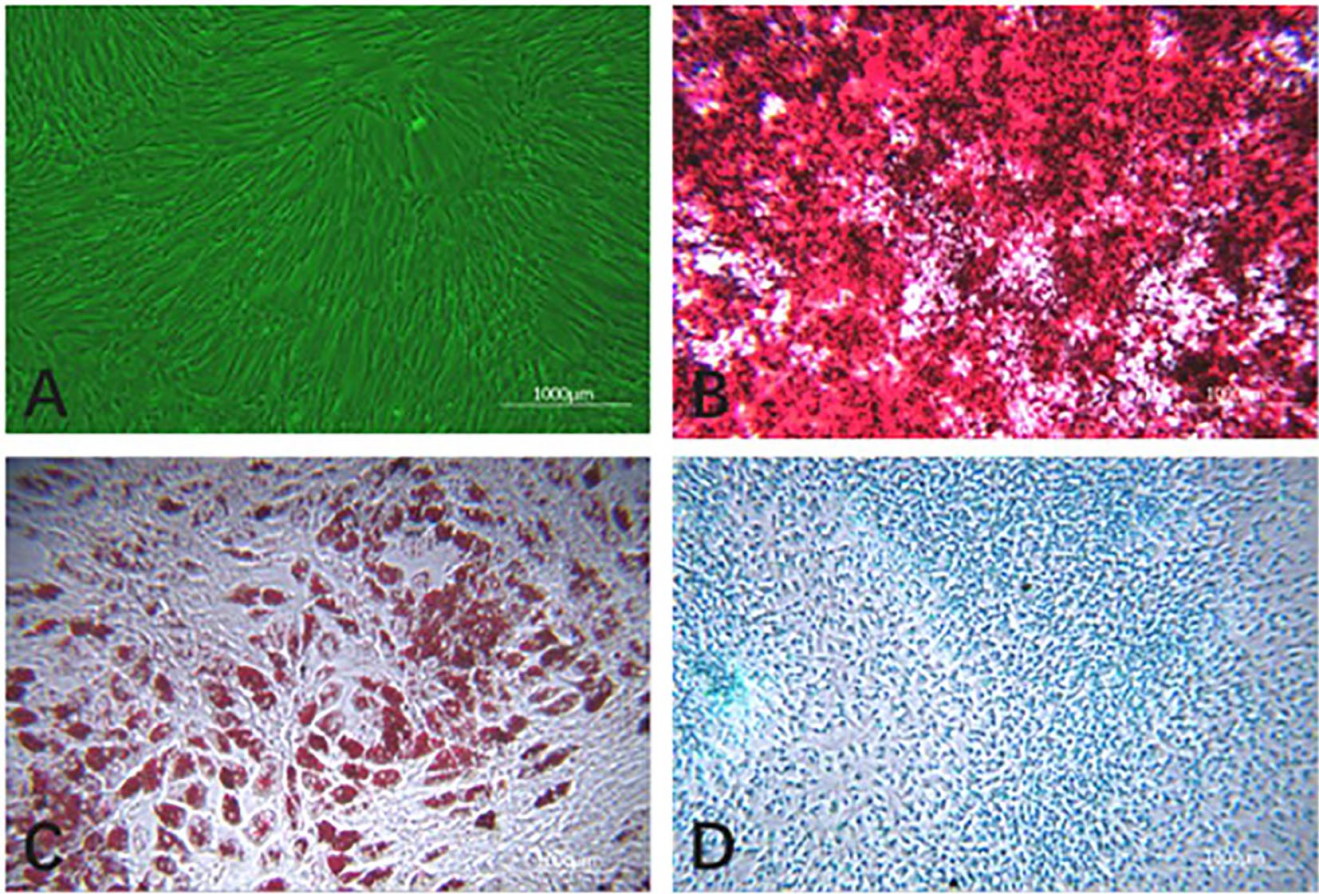
hUCMSC-induced differentiation results (× 400). (**A**) Shows uninduced hUCMSCs from the P5 generation; (**B**) shows osteogenic induction using alizarin red staining; (**C**) shows adipogenic induction after oil red O staining; (**D**) shows cartilage induction after staining with alcian blue.

HUCMSCs were induced to differentiate into bone, adipose tissue and cartilage (as shown in Fig. 8). The uninduced hUCMSC cell morphology did not change, and the cells presented as typical spindle-shaped, fibroblast-like cells (Fig. 8A). HUCMSCs induced to undergo osteogenesis in vitro exhibited a significant change in cell morphology, and a large number of calcium nodules appeared. Alizarin red staining showed a typical red color (Fig. 8B). HUCMSCs induced to undergo adipogenesis in vitro exhibited a cell morphology that showed shrinkage and rounding, and after oil red O staining, red droplets of fat were visible in the cells (Fig. 8C). HUCMSCs induced to undergo cartilage formation in vitro exhibited a significant change in morphology, and the endo-acid mucopolysaccharides in cartilage tissue showed obvious blue staining with alcian Blue (Fig. 8D).

#### Effect of the GFP labeling of hUCMSCs in vitro

**Figure 9:**
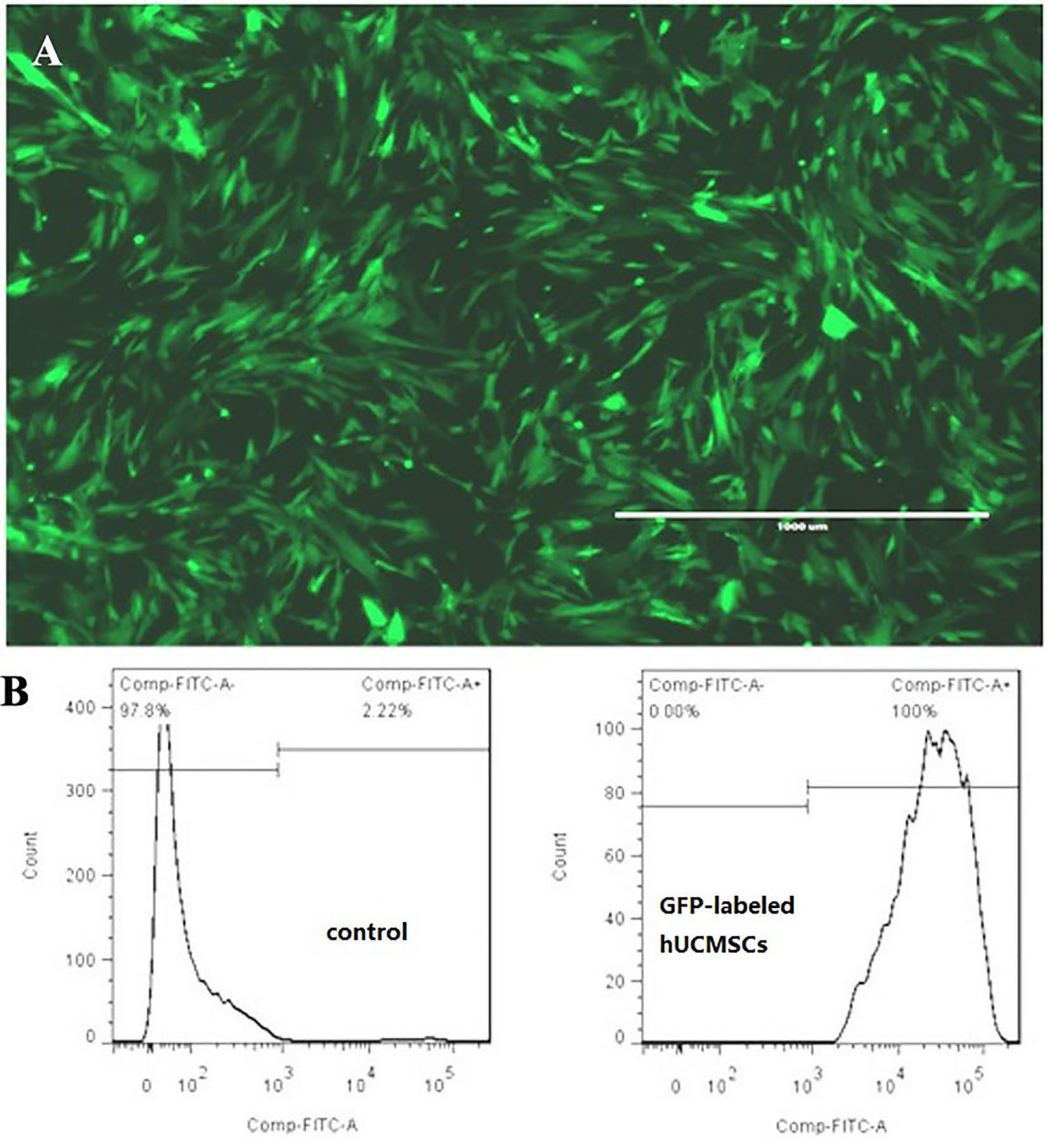
Effect of the GFP labeling of hUCMSCs in vitro. GFP-labeled hUCMSCs were observed under a fluorescence microscope, and the positive rate of GFP-labeled hUCMSCs was analyzed by flow cytometry. The positive rate after labeling was 100%.

The effect of GFP lentiviral transfection on hUCMSCs was observed under a fluorescence microscope (see Fig. 9A). It could be seen that the cells under the microscope showed a significant fluorescence response, and the transfection effect was good, indicating that the cells could be used for the in vivo tracking of experimental animals. Flow cytometry was used to analyze the positivity rate of this batch of GFP-labeled hUCMSCs (Fig. 9B). The green fluorescence response was obvious, and the positive labeling ratio was as high as 100.0%.

### Evaluation of the efficacy and mechanism of hUCMSCs

#### General status observation

**Figure 10:**
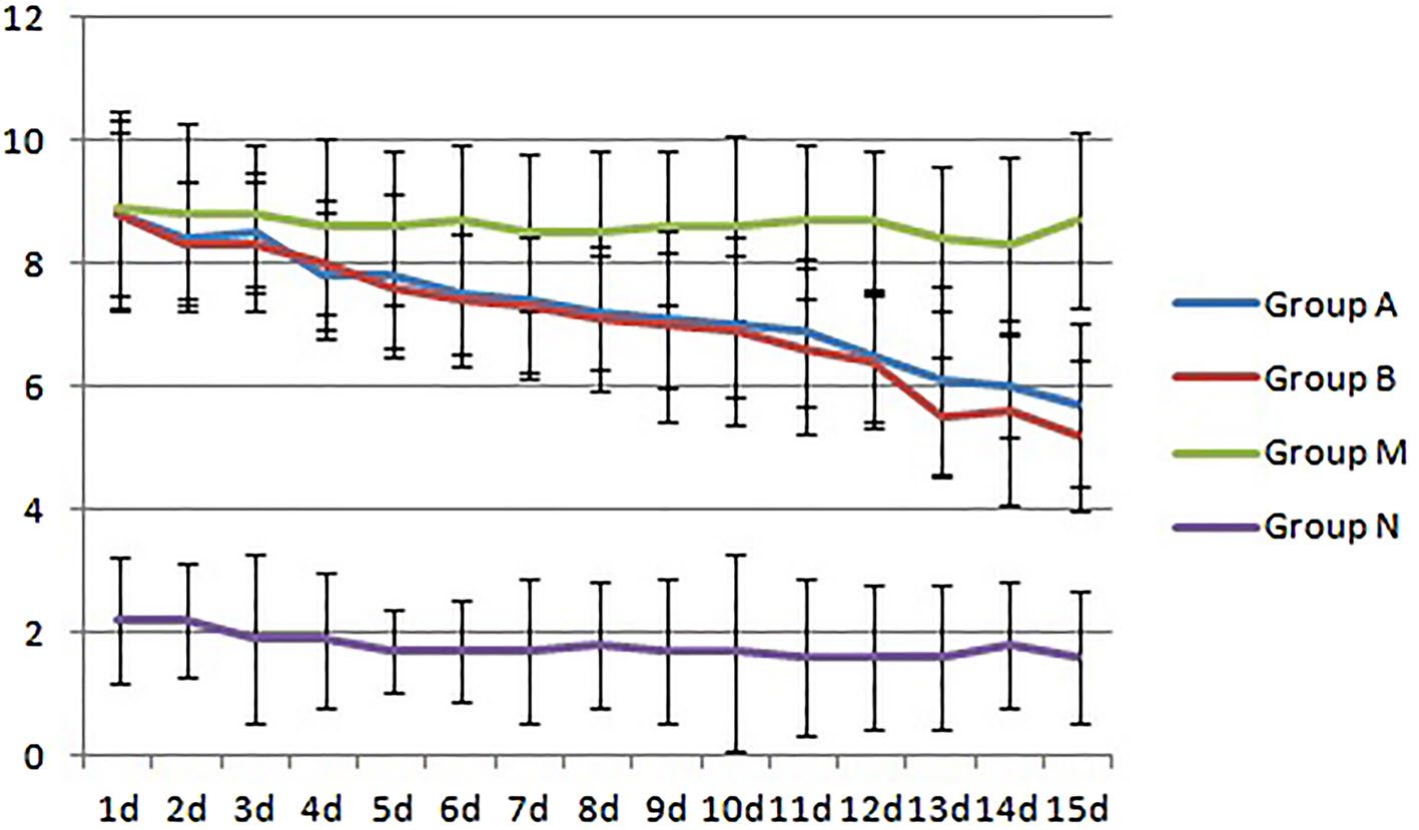
Symptom scores for the four groups. Group M was the model control group, for which the score was significantly higher than that of the healthy control group N (p < 0.05). Groups A and B were the model treatment groups. With the extension of the treatment time, the symptom score gradually decreased. Compared with the score of group M, there was a significant decrease in the score starting on the 4th day (p < 0.05), but the difference between the two groups was not obvious (p > 0.05).

From the first day of the mouse AR model treatment to the end of the treatment, the observation results of the general conditions, such as the occurrence of sneezing and runny nose in the mice in each group, were obtained (Fig. 10). The N group was the healthy control group, and the symptom score was always below 5 points; the M group was the model control group, and the symptom score continued to be approximately 8 points, which was significantly higher than that of the N group (p < 0.05). The A and B groups were the model treatment groups, and the symptom scores gradually decreased with the extension of the treatment time. Compared with that of the M group, the score began to decline significantly on the 4th day (p < 0.05) and was close to 5 points on the 15th day in the A and B groups, but the difference between the two groups was not significant (p > 0.05).

#### Structural changes of the nasal mucosa

**Figure 11:**
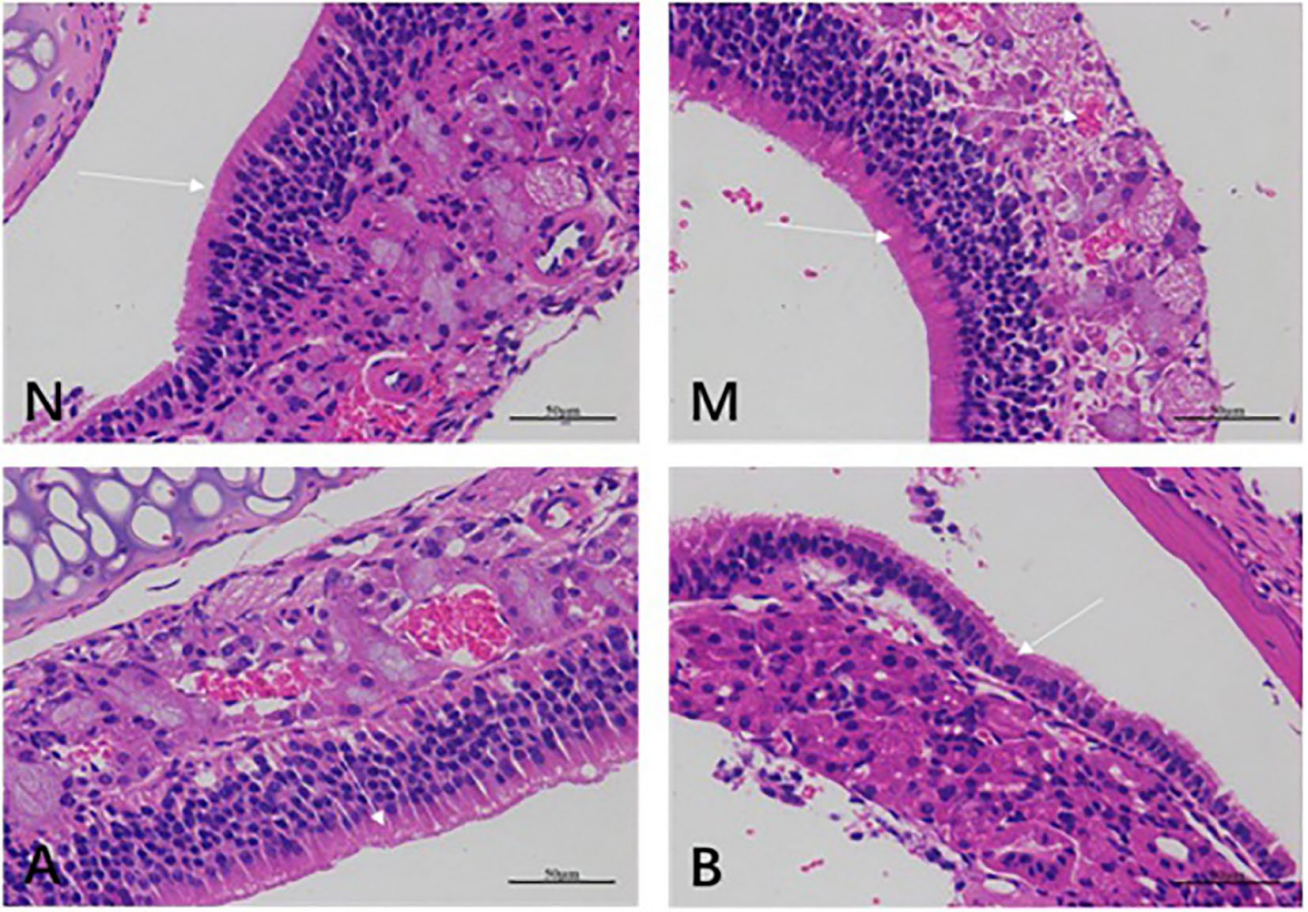
HE staining of the nasal mucosa of the four groups of mice (× 400). Group N consisted of healthy mice. Group M was the mouse AR model control group, which showed obvious vascular proliferation, as shown by the white arrows in the images. Groups A and B were the mouse AR model treatment groups. Groups B has more inflammatory cells than A group. It shows that the treatment effect of group A is better than group B.

The results of the conventional HE staining of nasal mucosa tissue sections of the four groups of mice are shown in Fig. 11. Group N consisted of healthy mice. The results showed that the nasal mucosa consisted of pseudostratified ciliated columnar epithelium, the cilia were intact, fewer blood vessels were visible, and there was no inflammatory cell infiltration (see Fig. 11N). Group M was the mouse AR model control group, and cilia shedding and lodging and the number of submucosal glands were increased; obvious inflammatory cell infiltration and obvious vascular proliferation, as shown by the white arrows in the picture, were also observed (see Fig. 11M). Groups A and B were the treated groups of the mouse AR model and showed a more complete ciliary structure and few infiltrating inflammatory cells; the blood vessels showed greater proliferation compared to those in the N group, but the degree of proliferation was far less obvious than that of the M group (see Fig. 11A,B).

#### Changes in total serum protein content in mice

**Figure 12:**
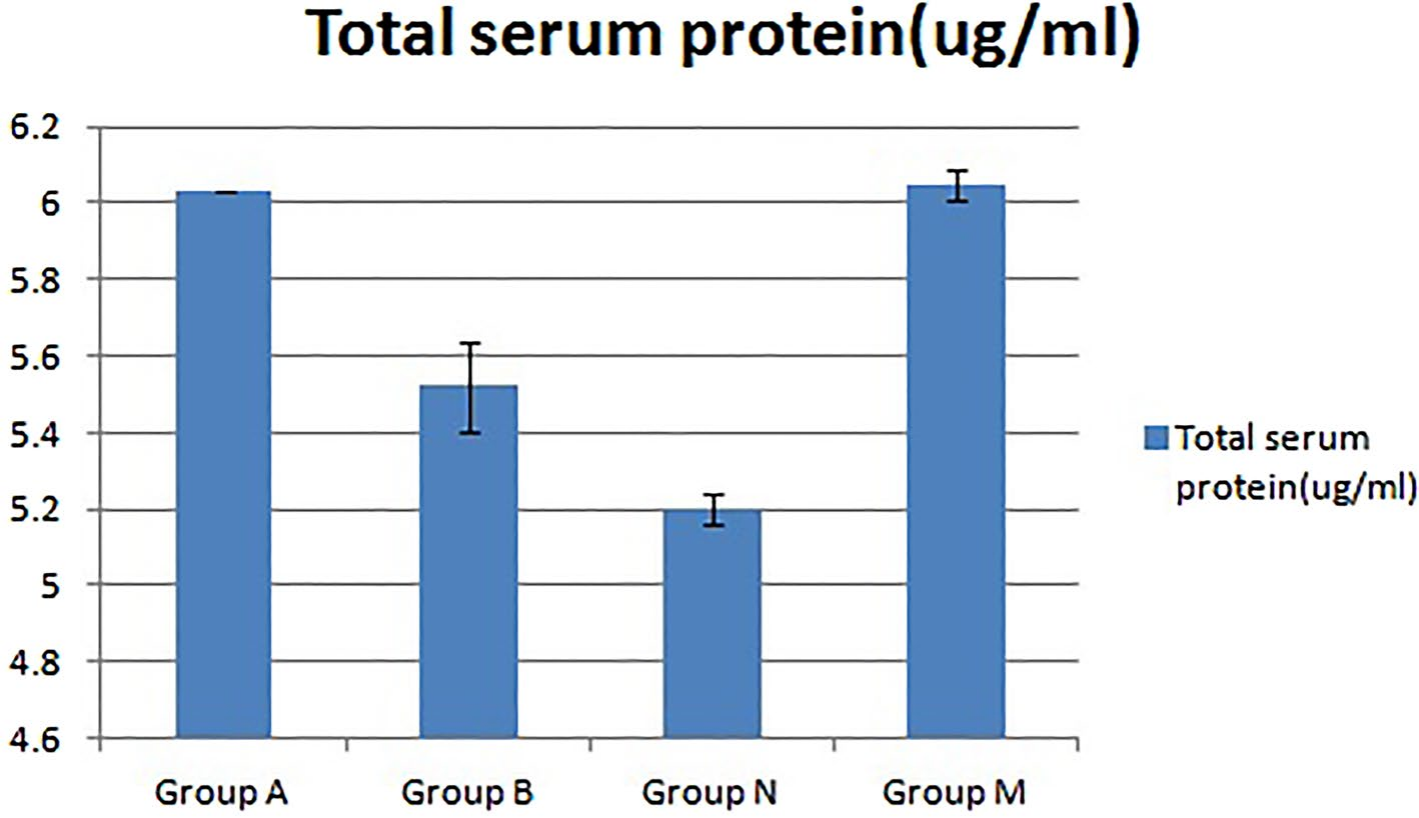
Changes in the serum total protein content of the four groups of mice (mean ± SD, n = 10). Compared with that in the healthy control group N, the total serum protein content in the A, B, and M groups was significantly increased (p < 0.05).

The BCA protein quantitative method was used to determine the total blood protein content in the peripheral blood of the four groups of mice (Fig. 12). Compared with that of group M, the total blood protein content of group A decreased slightly (p > 0.05), the total blood protein content of group B decreased more significantly (p < 0.05), and the differences between groups A and B were also large. Although the total peripheral blood protein content of groups A and B decreased, it was higher than that of group N.

#### Mouse serum IL-4 and INF-γ concentrations

**Figure 13:**
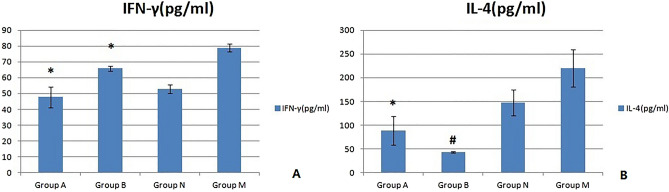
Serum IL-4 and INF-γ concentrations in the four groups of mice (mean ± SD, n = 10).

The ELISA results for the serum IFN-γ and IL-4 concentrations in the four groups of mice are shown in Fig. 13. Compared with those in group M, the concentrations of IFN-γ and IL-4 in the serum of mice in groups A and B were significantly decreased (*p < 0.05). In terms of the concentration of IFN-γ in serum, the decrease in group A was greater than that in group B. This decrease was significant (p < 0.05); for the serum IL-4 concentration, the decrease in group B was more significant than that in group A (^#^p < 0.01).

#### Detection of IL-4, IL-6, IL-10 and INF-γ mRNA transcript levels in mouse spleen

**Figure 14:**
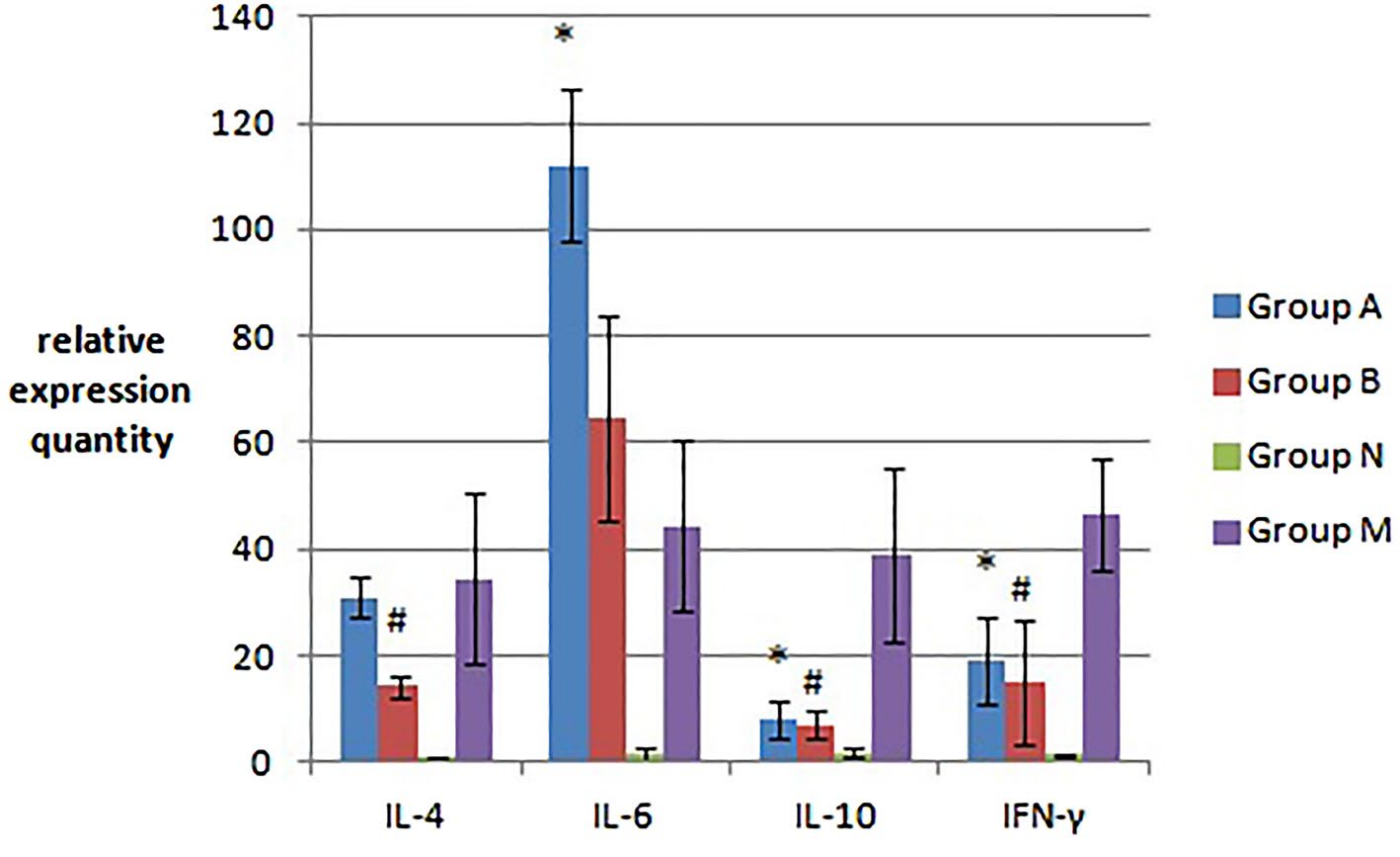
Detection of spleen IL-4, IL-6, IL-10 and INF-γ mRNA transcript levels in the four groups of mice (mean ± SD, n = 10). Compared with those in the spleen tissue of group M, the levels of IL-10 and IFN-γ mRNA in group A were significantly decreased (*p < 0.05), and the levels of IL-4, 10 and IFN-γ mRNA in group B were significantly decreased (^#^p < 0.05). There was significant increase in IL-6 in group A, compared to group M (*p < 0.05).

Quantitative real-time PCR was used to determine the transcription levels of IL-4, -6, and -10 and IFN-γ mRNA in the spleen of mice (Fig. 14). Compared with those in the spleen tissue of group M, the levels of IL-10 and IFN-γ mRNA in group A were significantly decreased (*p < 0.05), and the levels of IL-4 and -10 and IFN-γ mRNA in group B were significantly decreased (^#^p < 0.05). However, the transcription level of IL-4 mRNA in group A did not decrease significantly (p > 0.05), while the transcription level of IL-6 mRNA in the spleen tissue of mice in groups A and B increased, and the changes in group A were more significant (*p < 0.05); the IL-6 and -10 and IFN-γ mRNA transcript levels in the spleen of mice in each group were slightly higher in group A than in group B (p > 0.05), but the level of IL-4 mRNA transcription was significantly higher in group A than in group B (p < 0.05). The transcription levels of IL-4, -6, and -10 and IFN-γ mRNA in the spleen tissue of mice in each group were higher in groups A and B than those in group N (p < 0.05).

#### Tracking of GFP-labeled hUCMSCs in mice

**Figure 15:**
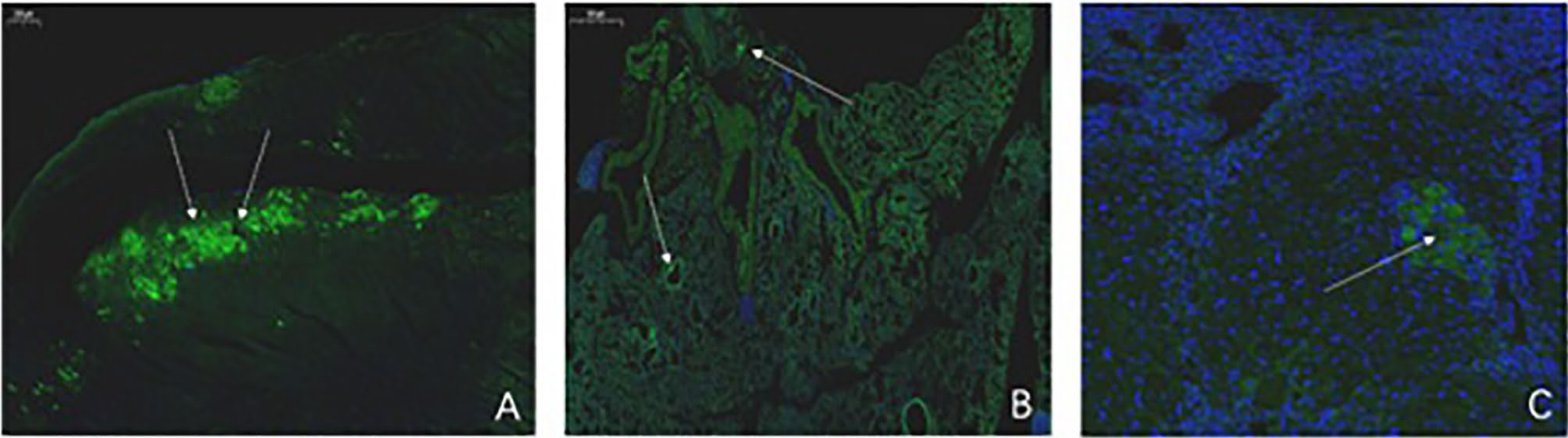
One week after transplanting GFP-labeled hUCMSCs, tissue sections were fluorescently imaged (× 50). (**A**) is heart tissue; (**B**) is lung tissue; (**C**) is ovarian tissue. From the picture, the positive cells in the heart are the most, and the lungs and ovaries are relatively few.

GFP-labeled hUCMSCs were transplanted into mice by different methods. One week after transplantation, samples were obtained from the animals. The heart, lung, and ovarian organs were stained with green fluorescent markers (Fig. 15). Different numbers of fluorescent cells were visible. The cells were successfully transplanted into mice.

**Figure 16:**
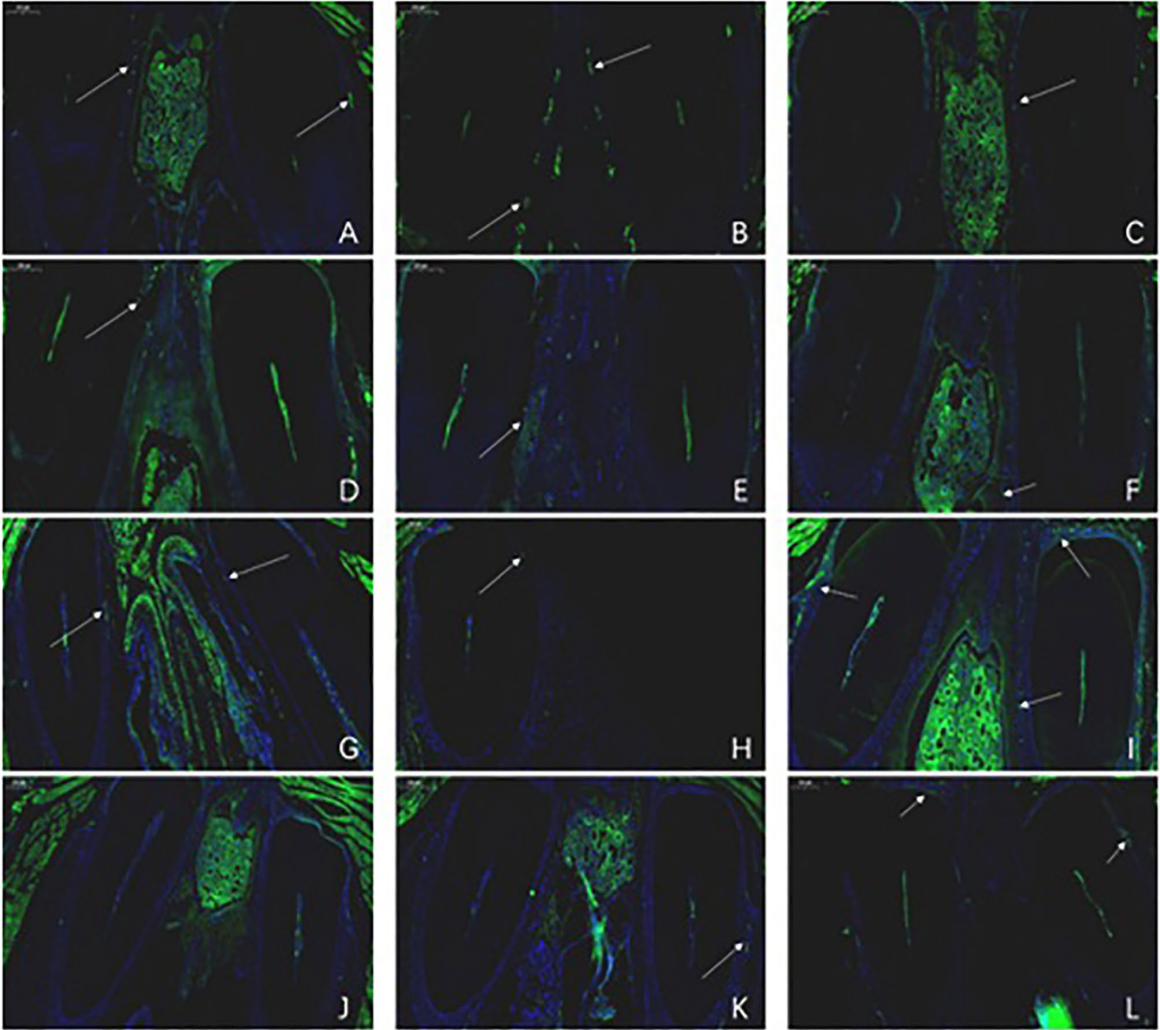
Fluorescent cell distribution (× 50) in nasal mucosa tissue sections obtained from mice on days 3, 7, 14, and 21 after transplantation of GFP-labeled hUCMSCs. (**A**–**C**) were obtained on the 3rd day, (**D**–**F**) were obtained on the 7th day, (**G**–**I**) were obtained on the 14th day, and (**J**–**L**) were obtained on the 21st day. (**A**), (**D**), (**G**), and (**J**) show the results of the nasal drip group; (**B**), (**E**), (**H**), and (**K**) show the results of the intraperitoneal injection group; (**C**), (**F**), (**I**), and (**L**) show the results of the tail vein injection group.

Tissue sections of nasal mucosa of mice from different time periods were single-labelled with green fluorescent markers, and the number of fluorescent cells in the nasal mucosa of mice was observed (Fig. 16). Green fluorescent cells could be seen in the nasal mucosa of the three groups of mice, and compared with that in other organs, the number was smaller. In the nasal drip group, a certain number of fluorescent cells was observed on the 3rd, 7th, and 14th days, and the number of fluorescent cells was close to zero on the 21st day (as shown in Fig. 16A,D,G,J). In the intraperitoneal injection group, fluorescent cells were visible in all time periods, and the number was the largest at week 2 and then gradually decreased (Fig. 16B,E,H,K). In the tail vein injection group, there were also a certain number of fluorescent cells in each time period. The number was the smallest on the third day and then gradually increased. The number of fluorescent cells was the largest in the second week, and the second-largest number was observed in the third week (Fig. 16C,F,I,L).

**Figure 17:**
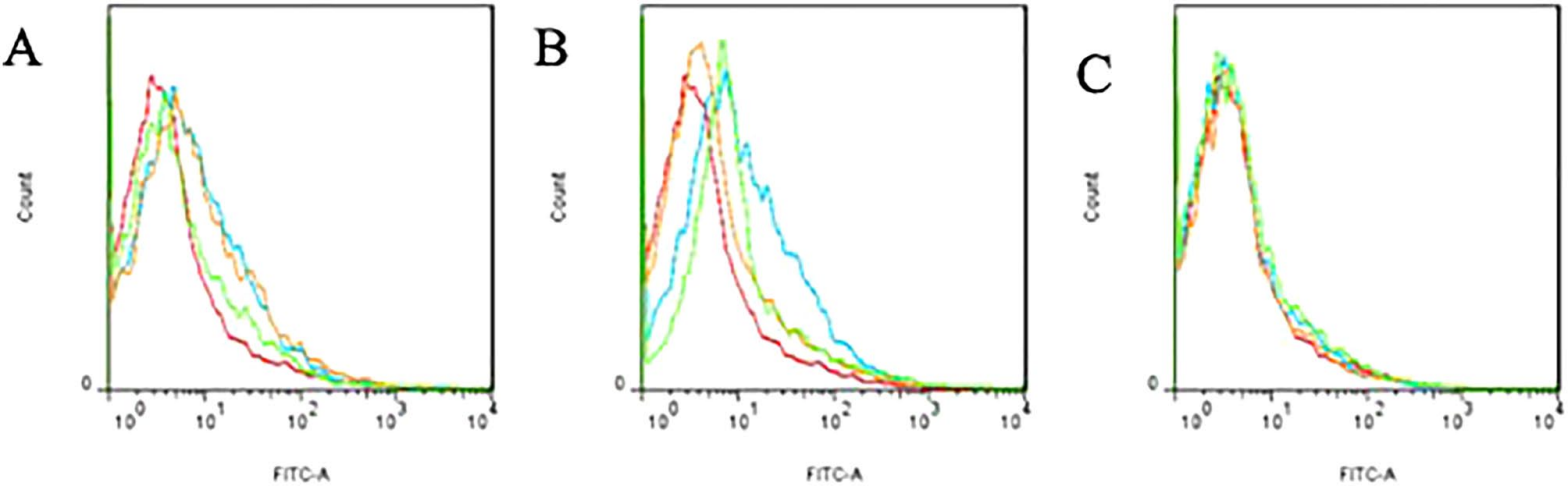
Flow cytometry analysis of the proportion of green fluorescent cells in the nasal mucosa of mice transplanted with GFP-labeled hUCMSCs on days 7, 14, and 21. (**A**) was obtained on day 7. (**B**) was obtained on day 14. (**C**) was obtained on day 21. The red line indicates the normal control group, the blue line indicates the nasal drip group, the orange line indicates the intraperitoneal injection group, and the green line indicates the tail vein injection group. By comparison with the blank group, it was found that the mice in each group had different proportions of fluorescent cells on the 7th and 14th days, with the most cells observed on the 14th day, but the proportion of fluorescent cells on the 21st day was close to zero.

Flow cytometry analysis of the proportion of green fluorescent cells in the nasal mucosa of mice at various time periods is shown in Fig. 17. By comparison with the blank group, it was found that the mice in each group had different proportions of fluorescent cells on the 7th and 14th days, with the most observed on the 14th day, but the proportion of fluorescent cells on the 21st day was close to zero.

## Discussion

### Diagnosis and treatment of AR

AR results in inflammation of the nasal mucosa, and AR is the most common disease causing mucosal inflammation^16^. Previously, AR was considered to be a disease confined to the nasal passages, but now it is considered to be a widespread systemic airway disease that is often accompanied by asthma. Because AR is a chronic disease, people often fail to diagnose it in time. Patients with cancer may have to seek medical treatment because they often recognize the serious impact it has on their daily lives, and AR is often considered by many to be an insignificant disease^17^. Therefore, when patients come to the hospital for treatment, doctors should pay special attention to checking the condition of the patient to obtain key medical histories and perform special physical examinations. As the most common atopic disease affecting 10–20% of the population, the diagnosis and treatment of AR deserves greater investigation. The clinical manifestations of AR are due to its pathophysiology as a result of a typical allergen reaction^18^. The most common allergens include dust mite stool particles, animal dander, mold and pollen. A history characterized by typical allergic symptoms is the basis for the diagnosis of allergic rhinitis. AR is diagnosed when two or more typical symptoms of AR, such as nasal congestion, nasal leakage, sneezing, and itching, persist for more than 1 h in most cases. Once the diagnosis of AR is confirmed through a combination of medical history, physical examination, and clinical examination, the stage of the disease must be appropriately determined, as this helps guide treatment. AR was previously classified as either seasonal (occurring at specific times of the year) or perennial (existing throughout the year); however, this method has now been replaced by a new classification method, and symptoms are classified as intermittent (less than 6 weeks) or persistent; persistent symptoms are classified as mild, moderate or severe according to the severity of the symptoms. Mild symptoms do not interfere with sleep or daily activities, while moderate to severe symptoms can cause sleep disturbances and work/life disorders. At present, although there are many treatment options, including oral drugs, topical sprays, and intranasal corticosteroids are the main treatment^19^, but they are not effective for some patients. In addition to basic symptoms such as nasal symptoms and nosebleeds, the use of these drugs is contraindicated in some patients, such as pregnant or nursing women. Moreover, AR is incurable. Regardless of what treatment method is adopted, the main goal is to reduce the symptoms of the disease as much as possible and improve the quality of life of patients. Therefore, it is necessary to develop new treatment schemes^20^.

### The establishment of AR animal models

Clinical studies of AR have not fully elucidated all aspects of the pathophysiology of disease. Animal models were established to better understand these mechanisms and to evaluate the safety and effectiveness of treatments before starting clinical trials^21^. Animal models are the easiest way to understand AR pathophysiology and help develop new treatments. A variety of animals have been used in experimental models, such as rats, guinea pigs, dogs, pigs, primates, and horses, and it is necessary to choose a suitable animal for allergy models^22^. Guinea pigs are often used as part of an AR model, since they are easy to manage and can be sensitized by inhalation of allergens and respiratory tract stimuli; however, there are immunological limitations. The most common species studied in the past two decades has been mice, especially BALB/c mice^23^. Mice are inexpensive. Most allergen-specific antigens are IgE produced as part of immune responses, and good genetic data is available for BALB/c mice^24^. AR animal models attempt to mimic the pathophysiology of human diseases, and they usually include two phases: sensitization and challenge. Allergic reactions are traditionally induced via intraperitoneal and subcutaneous routes, but intranasal allergens are increasingly used because AR is mostly caused by inhaled allergens. Allergens are administered by intranasal infusion of allergenic stimulants. The selection of allergens is another important issue for establishing a good animal model. Ovalbumin is a traditional sensitizer^25^ and can cause severe inflammation^26^. It is a good and readily available sensitizer. To enhance the effect of the sensitizer, it is generally necessary to add an immune adjuvant. Immune adjuvants are generally prepared with aluminum hydroxide, which has nontoxic characteristics and good adsorption capacity and can encapsulate the antigen at the injection site to avoid its clearance by the body. Studies have shown that the main function of aluminum hydroxide is to induce a humoral response and stimulate the body to produce a Th2-type response, thereby rapidly producing persistent IgE antibodies with a high degree of safety and good immune adjuvant properties.

In fact, during the modeling process, there are huge differences caused by the selection of various AR model animals, experimental protocols, and allergens, but few studies have evaluated these differences to determine the best model. Most recent studies have induced sensitization and challenge through the same pathways involved in human disease and the use of the same allergens that trigger clinical disease. However, many methods simply cannot reflect the processes involved in human disease. Researchers should think further about what the best approach is to better develop different AR models and to further advance the development of future treatments.

### Research status of hUCMSC immunoregulatory function

HUCMSCs have significant immunoregulatory capabilities^27^ and play an important role in both the innate and adaptive immune systems. In recent years, there has been increasing research on the role of hUCMSCs in the adaptive immune response. The interaction between hUCMSCs and immune cells is complex, and immune cells can be regulated by direct cell contact and secretion^28^, inhibiting their activity. The data show that hUCMSCs inhibit proliferation by inducing G0 blockade in the cell cycle in T cells, and they can also induce T cell apoptosis mediated by Fas-1-dependent pathways^29^. Under normal circumstances, hUCMSCs can promote the survival and proliferation of T lymphocytes through the IL-6-dependent pathway. However, after tissue damage, the immune system is activated, and T-cell-derived IFN-γ activates the immunoregulatory properties of hUCMSCs and begins to inhibit the activation and proliferation of immune cells. HUCMSCs then upregulate the expression of HGF, PD-L1, PGE2, and cyclooxygenase-2 to regulate immune function. Experiments have shown that more than 30 soluble factors are involved in the immunomodulatory activation and proliferation of hUCMSCs by T lymphocytes, including HGF, TGF-β, PGE2, nitric oxide, and IL-10. The immune microenvironment, which is composed of inflammatory cytokines, plays a key role in stimulating the innate and adaptive immune regulation of mesenchymal stem cells. Of course, direct contact with cells can also regulate immune cells. Cells expressing cell-surface immunosuppressive molecules, such as programmed death ligand 1 and Fas ligand 1, bind to immune cell surface receptors, resulting in the loss of immune cell function. There is evidence that hUCMSCs bind to activated immune cells, which keeps them in close contact and thereby enhances their immunosuppressive effects. Therefore, hUCMSCs play a central role in maintaining immune balance by interacting with cytokines, chemokines and cell surface molecules. Previous studies on hUCMSC immunoregulation have focused on the interaction between hUCMSCs and B lymphocytes, natural killer cells and dendritic cells^30^. In recent years, because hUCMSCs repair tissue damage and regulate the inflammatory response, increasing attention has been paid to the regulation of macrophages and T cells.

### Study of hUCMSC transplantation for disease treatment

HUCMSCs not only have pluripotent differentiation potential but also show strong immunoregulatory potential through interactions with T lymphocytes, B lymphocytes, natural killer cells and dendritic cells. The immune-regulating effect of hUCMSCs on the body makes them an ideal agent for the treatment of allergic diseases. By regulating the activation of immune cells, they suppress allergic reactions and thus control disease progression. In recent years, mesenchymal stem cells, including hUCMSCs, have been used in animal models or human clinical trials for the treatment of various diseases, such as AR, asthma^31^, osteoarthritis^32^, graft-related diseases such as anti-host disease (GVHD), multiple sclerosis, liver disease^33^, spinal cord injury^34^, diabetes^35^, and systemic lupus erythematosus^36–38^. Many studies have found that hUCMSCs play an important role in improving disease treatment by activating Treg cells and inhibiting the production of inflammatory cytokines in AR mouse models^39^, especially at the beginning of the disease. In addition, transplantation of mesenchymal stem cells that successfully differentiated into insulin-producing cells was able to correct streptozotocin-induced hyperglycemia in diabetic rodents and to improve survival of transplanted islets and has also been found to be beneficial for the treatment of noninsulin-dependent patients^40^. In addition, the transplantation of hUCMSCs by intranasal administration to treat Parkinson's disease^41^ was found to be able to smoothly adjust the key involved brain regions (such as the hippocampus and olfactory cortex) after 4 or 5 months of enzyme expression (by increasing tyrosine hydroxylase and reducing toxin 6-hydroxydopamine levels, for example), reducing damage to the ipsilateral striatum and substantia nigra^42^. In addition, hUCMSCs can enhance the cellular autophagy pathway, which is important in the removal of amyloid plaques^43^, activate Tregs to regulate the activation of microglia, and improve neuronal survival in vitro and in AD mouse models. Finally, hUCMSCs and fibronectin-immobilized PCL nanofibers were transplanted into an animal model of myocardial infarction with very good results^44^.

### Prospect of the clinical application of hUCMSCs

In fact, many phase I/II clinical trials have been completed to examine safety issues and the feasibility of the use of clinical stem cells for treating human diseases^45^. Several recent studies have reported that the clinical application of autologous and allogeneic hUCMSCs has not caused serious adverse reactions in some animal experiments. Therefore, the clinical application of hUCMSC therapy to human diseases will be possible in the near future. However, clinical application to many diseases is still subject to considerable research obstacles^46^, and the remaining challenges are mainly related to the safety and effectiveness of hUCMSCs, including the establishment of a sufficient and safe clinical-grade hUCMSC cell bank^47^ subject to strict quality control (such as the detection of cell surface-specific expression markers, cell viability, and endotoxins and carcinogenicity testing), determination of hUCMSC standards for treating various diseases (including cell concentration, transplantation method, transplantation time, etc.), and the in-depth exploration of the mechanism of hUCMSC treatment of various diseases. Furthermore, the long-term efficacy of the hUCMSC treatment of various diseases still needs to be tracked. Although these severe limitations need to be addressed for the use of hUCMSCs, the use of hUCMSCs in the clinic is still very promising because of the attractive advantages, including the anti-inflammatory and immunoregulatory functions of hUCMSCs. Therefore, more rigorous clinical trials and animal studies are needed to make hUCMSC treatment a safe and effective method for treating many diseases^48^.

## Conclusion

1. BALB/c mice were induced with OVA by intraperitoneal injection and nasal challenge. The AR mouse model was successfully generated. Through the comparison of various experimental results, it was found that treatment with the 25 µg OVA + 2 mg Al(OH)_3_ adjuvant/200 µl suspension had a better sensitization effect. The model has a good effect, the modeling method is simple and easy to perform, and the repeatability is high.
2. The hUCMSCs were obtained by the repeated direct adherence culture of tissue blocks. The cell morphology, growth characteristics, cell surface antigen expression, and ability to induce differentiation all met the standards, the operation was simple, and the growth rate was fast. GFP-labeled hUCMSCs have a very good effect, and the positive labeling rate can reach 100%; the cells are stable and can be used for animal tracking.
3. HUCMSC transplantation by intraperitoneal injection and tail vein injection has a certain effect on the AR mouse model. The comparison of the results shows that the tail vein injection group had a better effect; after transplantation of GFP-labeled hUCMSCs into mice, fluorescent cells could be seen in all major organs. The tracking of cells in the mouse nose through fluorescence microscopy and flow cytometry analysis found that the greatest number of nasal hUCMSCs was observed in the three groups of mice in the second week. This indicates that hUCMSCs can reach the nasal cavity, and treatment with hUCMSCs may inhibit the allergic response of the mouse AR model by inhibiting the expression of the cytokines IL-10 and INF-γ.

## Data Availability

All data are available in the main text.
